# Multifactorial Causes of Paranoid Schizophrenia With Auditory-Visual Hallucinations in a 31-Year-Old Male With History of Traumatic Brain Injury and Substance Abuse

**DOI:** 10.7759/cureus.25488

**Published:** 2022-05-30

**Authors:** Vincent Wong, Kyle Chin, Luba Leontieva

**Affiliations:** 1 Psychiatry, State University of New York Upstate Medical University, Syracuse, USA

**Keywords:** substance-related disorders, traumatic brain injury, alcohol use disorder, cannabis use disorder, tobacco use disorder, auditory-visual hallucinations, schizophrenia, paranoid schizophrenia, neurocognitive disorders

## Abstract

Schizophrenia is a chronic psychiatric disorder that classically presents with distortions of thought, behavior, and perceptions that are often misdiagnosed. One difficulty in diagnosing schizophrenia is due to its phenotypically heterogeneous condition that can be precipitated by a combination of genetic, epigenetic, and environmental factors. The prevalence of schizophrenia is roughly 1%, but it is often misdiagnosed. Possible differential diagnoses include depression or bipolar disorder with psychosis, psychosis due to a medical condition, schizotypal and schizoid personality disorders, and neurocognitive disorders.

In this case report, a 31-year-old male presents with thoughts of suicide following a recent exacerbation of his hallucinations. On presentation, the patient presented with a historical diagnosis of “paranoid schizophrenia” as well as a history of traumatic brain injury (TBI), poly-substance use disorder, and a family history of schizophrenia. This case serves to highlight the difficulties of making an accurate diagnosis and providing evidenced-based treatment.

## Introduction

Schizophrenia is a heterogeneous condition that can be precipitated by many genetic and epigenetic factors. It is hypothesized that the heritability of schizophrenia is roughly 24% to 55% and affects roughly 1% of the world’s population, affecting men, slightly more than women with a ratio of 1.4:1 [1]. For a typical presentation, symptoms usually occur in the late second to early third decade, however, later presentations have also been noted [1]. Schizophrenia clinically presents with positive symptoms and negative symptoms. Positive symptoms include lack of insight, hallucinations (auditory being the most common), delusions, disorganized thought/speech, and disorganized behaviors [2]. Negative symptoms include social withdrawal, self-neglect, loss of motivation and initiative, emotional blunting, and paucity of speech [2]. The most common positive symptoms that present clinically include auditory hallucinations, ideas of reference, delusions of reference, suspiciousness, delusional mood, delusions of persecution, thought alienation, and thoughts spoken aloud [2]. The mainstay of treatment includes psychopharmacology as well as psycho-education for patient and their family, psychotherapy, and coordinated team-based care [1].

Traumatic brain injury, psychosis, and schizophrenia

Traumatic brain injury (TBI) occurs when there is sudden damage to the brain and most commonly occurs as a result of a fall, motor vehicle accident, sports injuries, or assault [3]. Depending on the severity and location of the insult, the patient may develop psychosis secondary to TBI [3]. However, according to a meta-analysis by Fujii et al., psychotic disorder secondary to TBI (PDTBI) is difficult to diagnose given its vague criteria per the Diagnostic and Statistical Manual of Mental Disorders, fourth edition (DSM-IV) and attributing psychosis directly to prior TBI [3,4]. Furthermore, Fujii et al. found that there is a large range of latency following TBI and subsequent development of psychosis, with a mean delay of 4.1+ 6.6 years. Data regarding PDTBI also suggest that delusions are more common compared to hallucinations and that auditory hallucinations are the most common type of hallucination encountered [4]. The absence of negative symptoms also supports a diagnosis of PDTBI [4]. Atypical features of psychosis (e.g., atypical age of onset or non-auditory hallucinations) after a TBI can also suggest TBI-induced psychosis [5]. 

The pathophysiology underlying the relationship between TBI and psychosis is complex and seems to be influenced by individual-specific factors. For example, TBI may act as a stressor in a stress-diathesis model of schizophrenia [6]. In this model, a stressor i.e., TBI, interacting with underlying genetic susceptibility can precipitate psychosis. Studies on family history of schizophrenia as a risk factor for psychosis after TBI support this model [7,8]. Other studies suggest that TBI may directly impact structural and functional brain changes in sensory- and other information-processing networks; this can manifest with psychotic features such as hallucinations and delusions. Data that show lower rates of familial schizophrenia in patients with post-TBI psychosis than among patients with schizophrenia without TBI support this model of TBIs directly impacting structural and functional brain changes leading to schizophrenia [9,10].

Additionally in PDTBI, commonly found laboratory findings include abnormal findings on EEG (slowing followed by spiking as the most common abnormality found) and CT/MRI (most common findings localized to the frontal lobes, followed by the temporal lobes, and then the ventricles) [3]. With neuropsychological testing for patients with TBI, impairments to memory were the most common, followed by the executive and visuospatial functioning [3]. Comparatively, imaging studies (MRI) of patients with schizophrenia demonstrate a common finding of structural abnormalities within the frontotemporal and frontoparietal white matter tracts [11].

Substance abuse, psychosis, and schizophrenia

According to the DSM-V, substance-induced psychosis is a diagnosis of exclusion that is defined as symptoms of hallucinations or delusions arising within a month of substance intoxication or withdrawal. Specifically, cocaine, amphetamines, hallucinogens, and cannabis are known to elicit psychotic symptoms and are hypothesized to be related to the dopaminergic properties of the substance, either reducing its re-uptake or increasing its release [12]. Despite its clinical presentation being relatively similar, there are notable differences. Substance-induced psychosis has a later age of onset compared to schizophrenia [13]. Furthermore, greater antisocial personality traits, poor socioeconomic status, increased homelessness, and parental substance use were associated with substance-induced psychosis [13]. 

There are also well-researched relationships between schizophrenia and commonly abused substances such as alcohol, nicotine, cocaine, and cannabis. In particular, heavy cannabis abuse has been reported to be a stressor causing relapse in patients with schizophrenia [14]. The causal direction of the correlation between schizophrenia and cannabis use has been widely debated. Much evidence supports the hypothesis that cannabis use has a causal relationship that increases the risk for developing psychosis and schizophrenia, but some other hypotheses (specifically, schizophrenia increasing the use of cannabis, or the association is due to genetic pleiotropy) have also been suggested [15,16]. 

For a while it was presumed that the association between nicotine abuse and psychosis was due to reverse causation; and that smoking was secondary to psychosis as a form of self-medication, institutionalization, or by confounding by cannabis use and other social factors [17,18]. More recently, a bidirectional relationship has been proposed between cigarette smoking and psychosis; cigarette smoking may be causally related to the risk of psychosis, possibly via a shared genetic component between smoking and psychosis [17]. In one study, heavy smokers in monozygotic twin pairs were approximately 1.7-times more likely to develop psychosis compared with the nonsmoking twin thus demonstrating a possible shared genetic liability to both smoking and psychosis [17,18]. Although it is still debated as to the causal direction of the correlation between nicotine use and schizophrenia, it remains clear that there is an increased risk of schizophrenia in those with nicotine use disorder [17,18].

Just like cannabis use and nicotine use disorder, the causal direction of the correlation between alcohol abuse and schizophrenia is still debated and remains unclear as it is difficult to establish temporality between the diagnosis of schizophrenia and substance use disorder [19]. It is, however, established that there is an increased risk for developing schizophrenia in those with alcohol use disorder. In one study it was found that alcohol abuse increased the risk of developing schizophrenia by three times; however, a causal relationship has not yet been established [19]. 

## Case presentation

A 31-year-old Caucasian male with a reported past psychiatric history of paranoid schizophrenia, alcohol use disorder, tobacco use disorder, cannabis use disorder, depression, and a history of TBI was admitted to our inpatient psychiatric unit from the emergency department (ED) for a two-week history of worsening auditory/visual hallucinations as well as increased suicidal ideation. He also mentioned that he had just recently been discharged from another inpatient psychiatric unit recently and had been staying with his mother since then. The patient reported being noncompliant with his medication regimen, which was identified as baclofen 10mg bis in die or twice a day (BID), benztropine 1mg BID, fluoxetine 20mg, gabapentin 400mg BID, naltrexone 50mg, haloperidol 5mg per os or by mouth (PO) ter in die or three times a day (TID), haloperidol decanoate 100mg intramuscular (IM) (last administered a month before admission). He describes his hallucinations as hearing and seeing various objects and individuals, particularly at night. The patient also reported occasional cannabis and alcohol use but his urine drug screen and blood alcohol level on admission was negative. 

Regarding his psychiatric history, the patient was first diagnosed with long-standing depression at age 9 and was diagnosed with paranoid schizophrenia at age 26 during a 10-week inpatient psychiatric hospitalization. Since then, he reported multiple ED/comprehensive psychiatric emergency program (CPEP) and inpatient psychiatric admission as well as one suicide attempt in 2018, at age 29, when he attempted to slit his wrists. Although the patient had been established with various mental health follow-ups in the past, he denies any currently. 

The patient also reported a pertinent history of having a TBI when he was 20 years old due to a car accident in 2010. The patient had reported the loss of consciousness and had a Glasgow Coma Scale (GCS) score of 3 and blood alcohol of 0.24 when evaluated. Imaging at the time demonstrated a right frontal subdural hematoma with left midline shift as well as multiple bone fractures. The patient reported that he suffered extensive memory loss and had to relearn the majority of his physical and mental capacities. Subsequent neuropsychiatric testing demonstrated average intelligence with some impairments in aspects of attention as well as memory and learning for verbal/visual information. Furthermore, testing also demonstrated possible poor effort as well as significant mood difficulties (anxiety, depression). Montreal Cognitive Assessment (MoCA) was also administered and the patient scored 24/30, which indicates mild cognitive impairment.

The patient also had neuropsychiatric testing that was obtained one year following his TBI, which indicated that: 

”Mr. XXX is an individual with average intellect. His neuropsychological profile is notable for impairments in aspects of attention and learning and memory for non-contextualized bits of (most verbal) and visual information. All other performances were within the average range… Mr. XXX’s cognitive deficits on the exam could represent enduring sequel from his brain injury, his significant anxiety and depression, the variable effort he put forth on testing or a combination of these factors.”

**Figure 1:**
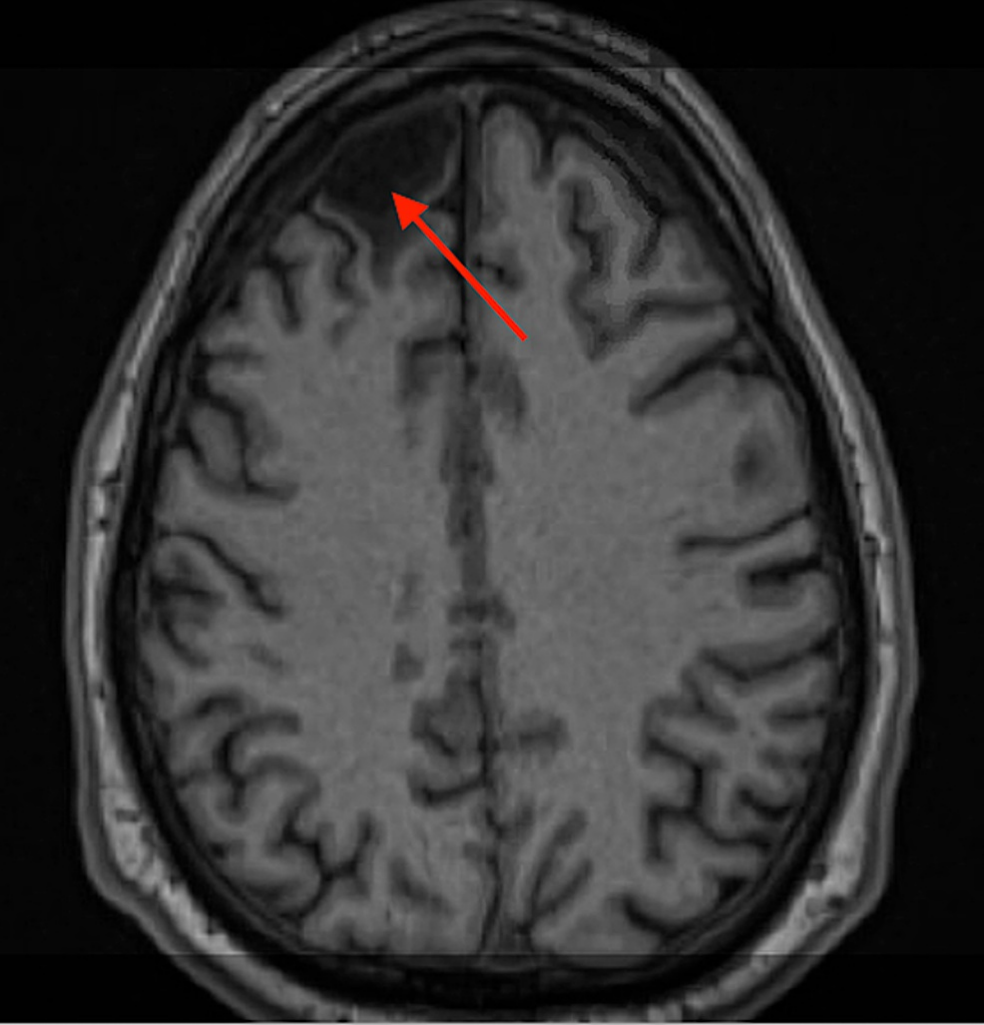
Redemonstrated cystic encephalomalacia in the right anterior frontal lobe with surrounding gliosis likely due to old trauma

Once in the unit, the patient reported seeing and hearing a balloon pop as well as seeing a suited, faceless, grey-scale individual later on in the day. However, he did not appear to be internally preoccupied and did not demonstrate any reactions to his reported hallucinations. Some psychomotor retardation as well as an impoverished thought process, and flat affect were also appreciated on examination. Given the patient’s history of TBI, an MRI of the brain was done, which demonstrated cystic encephalomalacia in the right anterior frontal lobe with surrounding gliosis, without any acute intracranial abnormalities (Figure 1). The encephalomalacia is most likely due to old trauma from his TBI. 

Collateral information was obtained from the patient’s mother who stated that he dropped out of college after the second semester, was in a severe car crash at age 20, and has been drinking since he was a teenager. The patient has also been smoking marijuana since the age of 17. He also has been smoking about one pack of cigarettes per day for 16 years. The patient's mother stated that the patient's father was on Haldol and possibly carried a diagnosis of schizophrenia, but is not certain. She also recalled observing some paranoia about her going to work and thinking that she was cheating on him. The patient’s mother also believes that the patient's aunt on her father's side had schizophrenia, but she was unable to elaborate on that. As far as addiction history, the mother states that there is alcohol addiction on both sides of the family. There are no suicide attempts on either side of the family. 

At the beginning of our patient's hospitalization, he endorsed audiovisual hallucinations (AVH) after stating that he missed taking his medications for three days about one week before his admission. He also endorsed passive suicidal ideation (SI) without intent or plan, mentioning that he was distressed by his hallucinations. He stated that his AVH primarily occur throughout the night. During his hospitalization, he was initially kept on his home medications. He tolerated these medications and denied any side effects; however, Haldol was held due to concerns of excessive sedation.

Throughout his hospitalization, the patient was very blunted and aloof. The patient was largely apathetic. Despite reporting some improvements in his AVH and mood, his presentation remained largely unchanged. Moreover, the patient did not appear to be a reliable historian, making several inconsistent comments about the nature of his audio hallucinations (AH) as well as the timeline of his symptoms. His fluoxetine was eventually increased to 40mg daily and he was restarted on haloperidol 5mg BID as needed which was eventually adjusted to 5mg nightly as he continued to endorse visual hallucinations (VH) throughout his hospitalization. He also received a haloperidol decanoate 100mg long-acting injection as he had previously been maintained on this medication as an outpatient. The patient also was prescribed 50mg of trazodone as well for insomnia. These medications were well tolerated and he reported that the haloperidol made him feel more relaxed. 

Later during his hospitalization, the patient's AVH diminished and he stated that he felt "good." He continued to deny SI and homicidal ideation (HI) and was without any physical complaints. The patient did not require any restraints or seclusions during his admission. He was in good behavioral control throughout hospitalization and attended both group and individual therapy. Throughout hospitalization, the patient reported improvement in his hallucinations. 

On the day of discharge, the patient stated his mood was "good." He denied any further symptoms of depression, mania, anxiety, and psychosis, including changes in sleep, mood, anhedonia, fatigue, anxiety, active/passive SI, HI, AH, and VH. The patient denied any side effects from medications, including headache, nausea/vomiting, rash, weight gain, muscle problems/akathisia/extrapyramidal side effects (EPS), and stated that the medications had helped. As such, he was discharged back to his mom and outpatient follow-up was scheduled five days following discharge from the inpatient psychiatry unit.

## Discussion

In this case, the patient identifies psychosis as a distressing symptom for him. Psychosis is a difficult symptom to manage given its varied etiology. In this case, our patient presents with a family history of treatment with haloperidol and presumed schizophrenia, a history of early exposure to substances (cannabis and alcohol), as well a history of severe brain trauma, which makes an accurate diagnosis and effective treatment difficult. However, there are several details that might help clarify a diagnosis.

In PDTBI, patients often display specific behavioral disturbances such as agitation and aggression [20]. Comorbid cognitive impairments, particularly involving attention, memory, and executive/frontal functions, are also common in post-TBI psychosis [4]. Additionally, patients tend to present with persecutory delusions. Hallucinations can also occur with post-TBI psychosis; the presence and type of hallucinations tend to be associated with the timing of onset of psychosis after TBI. Patients with later onset of symptoms (two years or longer after the trauma) were more likely to have hallucinations than early-onset patients (onset less than two years after trauma) [21]. 

Psychosis can also be precipitated by directly causing structural and functional changes in sensory- and other information-processing networks in the brain [1,9]. Data from different populations and methodologies, including imaging, EEG, and lesion location, consistently report abnormalities in the temporal areas being associated with PDTBI [20]. According to a meta-analysis performed by Fujii et al., 65% of patients who developed post-TBI psychosis had positive findings on CT/MRI with signs of atrophy and focal abnormalities most commonly in the frontal and temporal lobes for TBI-related psychosis [21]. In comparison, only 6% to 9% of patients with a primary psychotic disorder demonstrated focal findings and whose CT/MRI revealed enlarged ventricles and generalized atrophy [21]. Thus findings of focal lesions, especially in the frontal and temporal lobes can be a useful indicator. 

On a similar note, psychosis can also be precipitated by epileptic seizures [3]. Fuji et al. noted that 33% to 58% of patients who struggle with post-TBI psychosis also have a documented history of seizure disorders with positive EEG findings such as temporal slowing followed by spiking as the most common, with a significant majority of these abnormalities in the temporal lobes [21]. 

Additionally, neuropsychiatric testing may be useful to differentiate between post-TBI psychosis and primary psychosis. According to Fujii et al., it was found that 88% of cases reported impairments on neuropsychological testing. The most common impaired function was the memory, followed by executive and visuospatial functions [21]. 

Given the complex etiology of psychosis, the treatment of psychosis differs slightly based on its etiology. Guidelines recommend treating acute psychosis with an atypical antipsychotic, preferably aripiprazole or risperidone given their relatively favorable side effect profile [22]. Should treatment be ineffective, consider adding a second dose and achieving clinical improvements before tapering the first dose over a two-week period [22]. Other interventions such as family-based interventions and cognitive behavioral therapy (CBT) also help reduce distress related to psychosis [22]. Should treatment adherence be a concern, consider the use of long-acting injectable antipsychotics.

In schizophrenia, if negative symptoms appear to be more debilitating, data suggest that cariprazine is more effective [22]. Data also suggest the use of clozapine for treatment-resistant schizophrenia or persistent suicidality [23]. If a patient struggles with clozapine-resistant schizophrenia, consider augmentation with electroconvulsive therapy (ECT) (first-line) or augmenting with an atypical antipsychotic with aripiprazole being the most studied [23].

In a meta-analysis regarding pharmacological treatment of TBI neurobehavioral sequelae, data was organized into three levels of recommendations (standards, guidelines, and options), with standards having the most evidence and options having the least. Given the lack of sufficient high-powered data as such, several options such as amitriptyline, desipramine, and sertraline were recommended for the treatment of major depressive disorder (MDD), and olanzapine for psychosis) [24]. There is enough evidence to support guidelines for the use of methylphenidate and donepezil for deficits in attention/processing [24]. Similarly, the use of donepezil for memory and bromocriptine for deficits in executive functioning also have enough evidence to support for them being guideline recommendations [24]. 

The location of the patient’s cystic encephalomalacia in the right anterior frontal lobe region, the timing of onset of hallucinations, and results from his neuropsychiatric testing seem to indicate post-TBI psychosis. However, schizophrenia cannot be ruled out at this time given family history and historical diagnosis of schizophrenia. While hospitalized at the inpatient psychiatric unit, he did not appear internally preoccupied with reported visual hallucinations, but would often report feeling distressed. This might be related to poor stress tolerance and difficulty in emotional processing. However, another plausible explanation is negative symptoms related to schizophrenia (affective flattening, alogia, and avolition). 

As schizophrenia cannot be ruled out, treatment with an antipsychotic appears to be indicated. Although haloperidol decanoate was selected in this case, given the history of TBI as well as possible negative symptoms, the patient might have had more success with an atypical antipsychotic such as aripiprazole and risperidone/paliperidone, given the availability and favorable side effect profile of long-acting injectable formulation. Although the patient’s rationale for his substance use was not thoroughly assessed, many patients with schizophrenia use substances to alleviate these extrapyramidal side effects [18], which also support switching to an atypical antipsychotic. Patients would also benefit from motivational enhancement interventions, which can refine skills and decrease substance use [6]. As for concerns of emotional distress, our patient might also benefit from optimizing his fluoxetine for the treatment of underlying depression. 

Another possible area of treatment is our patient's cognitive deficits in aspects of attention, learning, and memory, as evidenced by the MoCA score of 24/30 and prior neuropsychological testing. As such, the patient might have benefited from treatment with donepezil [24]. Similarly, he may also benefit from increased services including, but not limited to neurology, psychiatry, sleep medicine, rehabilitation services, and social work depending on the nature and mechanism of the injuries [25]. Additionally, there is evidence that early specialized multidisciplinary neuro-rehabilitation treatment relative to onset of injury has beneficial effects on recovery [26]. 

## Conclusions

Psychosis is often a symptom of many various etiologies, from primary thought disorders to medical conditions such as TBIs, structural brain damage, seizures, and substance intoxication/withdrawal amongst others. Although initial management involves the use of antipsychotics, clarifying its etiology is fundamental for successful treatment. With our patient, the development of psychosis was multifactorial: family history of schizophrenia, structural brain damage due to TBI, and a history of polysubstance use. Given such confounding factors, the use of EEG, brain imaging (CT/MRI), and neuropsychiatric testing provided valuable information to narrow the differential diagnoses. In this case, the patient is suspected to have post-TBI-related psychosis given the timeline of symptoms, location of cystic encephalomalacia in the right anterior frontal lobe, and results of his neuropsychiatric testing. However, schizophrenia cannot be ruled out. As such, recovery can be optimized through a comprehensive and early multidisciplinary approach targeting rehabilitation. 

## References

[1] Chen L, Selvendra A, Stewart A, Castle D (2018). Risk factors in early and late onset schizophrenia. Compr Psychiatry.

[2] Picchioni MM, Murray RM (2007). Schizophrenia. BMJ.

[3] Fujii D, Ahmed I (2010). The spectrum of psychotic disorders: neurobiology, etiology and pathogenesis.

[4] Fujii D, Ahmed I (2002). Psychotic disorder following traumatic brain injury: a conceptual framework. Cogn Neuropsychiatry.

[5] Arciniegas DB, Harris SN, Brousseau KM (2003). Psychosis following traumatic brain injury. Int Rev Psychiatry.

[6] Jones SR, Fernyhough C (2007). A new look at the neural diathesis--stress model of schizophrenia: the primacy of social-evaluative and uncontrollable situations. Schizophr Bull.

[7] Malaspina D, Goetz RR, Friedman JH (2001). Traumatic brain injury and schizophrenia in members of schizophrenia and bipolar disorder pedigrees. Am J Psychiatry.

[8] Sachdev P, Smith JS, Cathcart S (2001). Schizophrenia-like psychosis following traumatic brain injury: a chart-based descriptive and case-control study. Psychol Med.

[9] Buckley P, Stack JP, Madigan C (1993). Magnetic resonance imaging of schizophrenia-like psychoses associated with cerebral trauma: clinicopathological correlates. Am J Psychiatry.

[10] Davison K (1983). Schizophrenia-like psychoses associated with organic cerebral disorders: a review. Psychiatr Dev.

[11] Burns J, Job D, Bastin M (2003). Structural disconnectivity in schizophrenia: a diffusion tensor magnetic resonance imaging study. British Journal of Psychiatry.

[12] Fiorentini A, Volonteri LS, Dragogna F, Rovera C, Maffini M, Mauri MC, Altamura CA (2011). Substance-induced psychoses: a critical review of the literature. Curr Drug Abuse Rev.

[13] Caton CL, Drake RE, Hasin DS, Dominguez B, Shrout PE, Samet S, Schanzer B (2005). Differences between early-phase primary psychotic disorders with concurrent substance use and substance-induced psychoses. Arch Gen Psychiatry.

[14] Winklbaur B, Ebner N, Sachs G, Thau K, Fischer G (2006). Substance abuse in patients with schizophrenia. Dialogues Clin Neurosci.

[15] Pasman JA, Verweij KJ, Gerring Z (2018). GWAS of lifetime cannabis use reveals new risk loci, genetic overlap with psychiatric traits, and a causal influence of schizophrenia. Nat Neurosci.

[16] Vaucher J, Keating BJ, Lasserre AM (2018). Cannabis use and risk of schizophrenia: a Mendelian randomization study. Mol Psychiatry.

[17] Kendler KS, Lönn SL, Sundquist J, Sundquist K (2015). Smoking and schizophrenia in population cohorts of Swedish women and men: a prospective co-relative control study. Am J Psychiatry.

[18] Quigley H, MacCabe JH (2019). The relationship between nicotine and psychosis. Ther Adv Psychopharmacol.

[19] Nielsen SM, Toftdahl NG, Nordentoft M, Hjorthøj C (2017). Association between alcohol, cannabis, and other illicit substance abuse and risk of developing schizophrenia: a nationwide population based register study. Psychol Med.

[20] Schwarzbold M, Diaz A, Martins ET (2008). Psychiatric disorders and traumatic brain injury. Neuropsychiatr Dis Treat.

[21] Fujii D, Ahmed I (2002). Characteristics of psychotic disorder due to traumatic brain injury: an analysis of case studies in the literature. J Neuropsychiatry Clin Neurosci.

[22] Marder Marder, S S (2022). Psychosis in adults, initial management. UpToDate.

[23] Stroup T, Marder S (2022). Schizophrenia in adults: maintenance therapy and side effect management. https://www.uptodate.com/contents/schizophrenia-in-adults-maintenance-therapy-and-side-effect-management.

[24] Warden DL, Gordon B, McAllister TW (2006). Guidelines for the pharmacologic treatment of neurobehavioral sequelae of traumatic brain injury. J Neurotrauma.

[25] Morse AM, Garner DR (2018). Traumatic brain injury, sleep disorders, and psychiatric disorders: an underrecognized relationship. Med Sci.

[26] Königs M, Beurskens EA, Snoep L, Scherder EJ, Oosterlaan J (2018). Effects of timing and intensity of neurorehabilitation on functional outcome after traumatic brain injury: a systematic review and meta-analysis. Arch Phys Med Rehabil.

